# Measurement of the average very forward energy as a function of the track multiplicity at central pseudorapidities in proton-proton collisions at $\sqrt{s}=13\,\text {TeV}$

**DOI:** 10.1140/epjc/s10052-019-7402-3

**Published:** 2019-11-05

**Authors:** A. M. Sirunyan, A. Tumasyan, W. Adam, F. Ambrogi, T. Bergauer, J. Brandstetter, M. Dragicevic, J. Erö, A. Escalante Del Valle, M. Flechl, R. Frühwirth, M. Jeitler, N. Krammer, I. Krätschmer, D. Liko, T. Madlener, I. Mikulec, N. Rad, J. Schieck, R. Schöfbeck, M. Spanring, D. Spitzbart, W. Waltenberger, J. Wittmann, C.-E. Wulz, M. Zarucki, V. Drugakov, V. Mossolov, J. Suarez Gonzalez, M. R Darwish, E. A. De Wolf, D. Di Croce, X. Janssen, J. Lauwers, A. Lelek, M. Pieters, H. Van Haevermaet, P. Van Mechelen, N. Van Remortel, F. Blekman, S. S. Chhibra, J. D’Hondt, J. De Clercq, G. Flouris, D. Lontkovskyi, S. Lowette, I. Marchesini, S. Moortgat, L. Moreels, Q. Python, K. Skovpen, S. Tavernier, W. Van Doninck, P. Van Mulders, I. Van Parijs, D. Beghin, B. Bilin, H. Brun, B. Clerbaux, G. De Lentdecker, H. Delannoy, B. Dorney, L. Favart, A. Grebenyuk, A. K. Kalsi, J. Luetic, A. Popov, N. Postiau, E. Starling, L. Thomas, C. Vander Velde, P. Vanlaer, D. Vannerom, Q. Wang, T. Cornelis, D. Dobur, A. Fagot, M. Gul, I. Khvastunov, C. Roskas, D. Trocino, M. Tytgat, W. Verbeke, B. Vermassen, M. Vit, N. Zaganidis, O. Bondu, G. Bruno, C. Caputo, P. David, C. Delaere, M. Delcourt, A. Giammanco, G. Krintiras, V. Lemaitre, A. Magitteri, K. Piotrzkowski, J. Prisciandaro, A. Saggio, M. Vidal Marono, P. Vischia, J. Zobec, F. L. Alves, G. A. Alves, G. Correia Silva, C. Hensel, A. Moraes, P. Rebello Teles, E. Belchior Batista Das Chagas, W. Carvalho, J. Chinellato, E. Coelho, E. M. Da Costa, G. G. Da Silveira, D. De Jesus Damiao, C. De Oliveira Martins, S. Fonseca De Souza, L. M. Huertas Guativa, H. Malbouisson, D. Matos Figueiredo, M. Medina Jaime, M. Melo De Almeida, C. Mora Herrera, L. Mundim, H. Nogima, W. L. Prado Da Silva, L. J. Sanchez Rosas, A. Santoro, A. Sznajder, M. Thiel, E. J. Tonelli Manganote, F. Torres Da Silva De Araujo, A. Vilela Pereira, S. Ahuja, C. A. Bernardes, L. Calligaris, D. De Souza Lemos, T. R. Fernandez Perez Tomei, E. M. Gregores, P. G. Mercadante, S. F. Novaes, SandraS. Padula, A. Aleksandrov, G. Antchev, R. Hadjiiska, P. Iaydjiev, A. Marinov, M. Misheva, M. Rodozov, M. Shopova, G. Sultanov, A. Dimitrov, L. Litov, B. Pavlov, P. Petkov, W. Fang, X. Gao, L. Yuan, M. Ahmad, G. M. Chen, H. S. Chen, M. Chen, C. H. Jiang, D. Leggat, H. Liao, Z. Liu, S. M. Shaheen, A. Spiezia, J. Tao, E. Yazgan, H. Zhang, S. Zhang, J. Zhao, A. Agapitos, Y. Ban, G. Chen, A. Levin, J. Li, L. Li, Q. Li, Y. Mao, S. J. Qian, D. Wang, Y. Wang, C. Avila, A. Cabrera, L. F. Chaparro Sierra, C. Florez, C. F. González Hernández, M. A. Segura Delgado, J. D. Ruiz Alvarez, D. Giljanović, N. Godinovic, D. Lelas, I. Puljak, T. Sculac, Z. Antunovic, M. Kovac, V. Brigljevic, D. Ferencek, K. Kadija, B. Mesic, M. Roguljic, A. Starodumov, T. Susa, M. W. Ather, A. Attikis, E. Erodotou, A. Ioannou, M. Kolosova, S. Konstantinou, G. Mavromanolakis, J. Mousa, C. Nicolaou, F. Ptochos, P. A. Razis, H. Rykaczewski, D. Tsiakkouri, M. Finger, M. Finger, E. Ayala, E. Carrera Jarrin, M. A. Mahmoud, Y. Mohammed, S. Bhowmik, A. Carvalho Antunes De Oliveira, R. K. Dewanjee, K. Ehataht, M. Kadastik, M. Raidal, C. Veelken, P. Eerola, H. Kirschenmann, K. Osterberg, J. Pekkanen, M. Voutilainen, F. Garcia, J. Havukainen, J. K. Heikkilä, T. Järvinen, V. Karimäki, R. Kinnunen, T. Lampén, K. Lassila-Perini, S. Laurila, S. Lehti, T. Lindén, P. Luukka, T. Mäenpää, H. Siikonen, E. Tuominen, J. Tuominiemi, T. Tuuva, M. Besancon, F. Couderc, M. Dejardin, D. Denegri, B. Fabbro, J. L. Faure, F. Ferri, S. Ganjour, A. Givernaud, P. Gras, G. Hamel de Monchenault, P. Jarry, C. Leloup, E. Locci, J. Malcles, J. Rander, A. Rosowsky, M. Ö. Sahin, A. Savoy-Navarro, M. Titov, C. Amendola, F. Beaudette, P. Busson, C. Charlot, B. Diab, R. Granier de Cassagnac, I. Kucher, A. Lobanov, C. Martin Perez, M. Nguyen, C. Ochando, P. Paganini, J. Rembser, R. Salerno, J. B. Sauvan, Y. Sirois, A. Zabi, A. Zghiche, J.-L. Agram, J. Andrea, D. Bloch, G. Bourgatte, J.-M. Brom, E. C. Chabert, C. Collard, E. Conte, J.-C. Fontaine, D. Gelé, U. Goerlach, M. Jansová, A.-C. Le Bihan, N. Tonon, P. Van Hove, S. Gadrat, S. Beauceron, C. Bernet, G. Boudoul, C. Camen, N. Chanon, R. Chierici, D. Contardo, P. Depasse, H. El Mamouni, J. Fay, S. Gascon, M. Gouzevitch, B. Ille, Sa. Jain, F. Lagarde, I. B. Laktineh, H. Lattaud, M. Lethuillier, L. Mirabito, S. Perries, V. Sordini, G. Touquet, M. Vander Donckt, S. Viret, A. Khvedelidze, Z. Tsamalaidze, C. Autermann, L. Feld, M. K. Kiesel, K. Klein, M. Lipinski, D. Meuser, A. Pauls, M. Preuten, M. P. Rauch, C. Schomakers, J. Schulz, M. Teroerde, B. Wittmer, A. Albert, M. Erdmann, S. Erdweg, T. Esch, B. Fischer, R. Fischer, S. Ghosh, T. Hebbeker, K. Hoepfner, H. Keller, L. Mastrolorenzo, M. Merschmeyer, A. Meyer, P. Millet, G. Mocellin, S. Mondal, S. Mukherjee, D. Noll, A. Novak, T. Pook, A. Pozdnyakov, T. Quast, M. Radziej, Y. Rath, H. Reithler, M. Rieger, A. Schmidt, S. C. Schuler, A. Sharma, S. Thüer, S. Wiedenbeck, G. Flügge, O. Hlushchenko, T. Kress, T. Müller, A. Nehrkorn, A. Nowack, C. Pistone, O. Pooth, D. Roy, H. Sert, A. Stahl, M. Aldaya Martin, C. Asawatangtrakuldee, P. Asmuss, I. Babounikau, H. Bakhshiansohi, K. Beernaert, O. Behnke, U. Behrens, A. Bermúdez Martínez, D. Bertsche, A. A. Bin Anuar, K. Borras, V. Botta, A. Campbell, A. Cardini, P. Connor, S. Consuegra Rodríguez, C. Contreras-Campana, V. Danilov, A. De Wit, M. M. Defranchis, C. Diez Pardos, D. Domínguez Damiani, G. Eckerlin, D. Eckstein, T. Eichhorn, A. Elwood, E. Eren, E. Gallo, A. Geiser, J. M. Grados Luyando, A. Grohsjean, M. Guthoff, M. Haranko, A. Harb, N. Z. Jomhari, H. Jung, A. Kasem, M. Kasemann, J. Keaveney, C. Kleinwort, J. Knolle, D. Krücker, W. Lange, T. Lenz, J. Leonard, J. Lidrych, K. Lipka, W. Lohmann, R. Mankel, I.-A. Melzer-Pellmann, A. B. Meyer, M. Meyer, M. Missiroli, G. Mittag, J. Mnich, A. Mussgiller, V. Myronenko, D. Pérez Adán, S. K. Pflitsch, D. Pitzl, A. Raspereza, A. Saibel, M. Savitskyi, V. Scheurer, P. Schütze, C. Schwanenberger, R. Shevchenko, A. Singh, H. Tholen, O. Turkot, A. Vagnerini, M. Van De Klundert, G. P. Van Onsem, R. Walsh, Y. Wen, K. Wichmann, C. Wissing, O. Zenaiev, R. Zlebcik, R. Aggleton, S. Bein, L. Benato, A. Benecke, V. Blobel, T. Dreyer, A. Ebrahimi, A. Fröhlich, C. Garbers, E. Garutti, D. Gonzalez, P. Gunnellini, J. Haller, A. Hinzmann, A. Karavdina, G. Kasieczka, R. Klanner, R. Kogler, N. Kovalchuk, S. Kurz, V. Kutzner, J. Lange, T. Lange, A. Malara, D. Marconi, J. Multhaup, M. Niedziela, C. E. N. Niemeyer, D. Nowatschin, A. Perieanu, A. Reimers, O. Rieger, C. Scharf, P. Schleper, S. Schumann, J. Schwandt, J. Sonneveld, H. Stadie, G. Steinbrück, F. M. Stober, M. Stöver, B. Vormwald, I. Zoi, M. Akbiyik, C. Barth, M. Baselga, S. Baur, T. Berger, E. Butz, R. Caspart, T. Chwalek, W. De Boer, A. Dierlamm, K. El Morabit, N. Faltermann, M. Giffels, P. Goldenzweig, M. A. Harrendorf, F. Hartmann, U. Husemann, S. Kudella, S. Mitra, M. U. Mozer, Th. Müller, M. Musich, A. Nürnberg, G. Quast, K. Rabbertz, M. Schröder, I. Shvetsov, H. J. Simonis, R. Ulrich, M. Weber, C. Wöhrmann, R. Wolf, G. Anagnostou, P. Asenov, G. Daskalakis, T. Geralis, A. Kyriakis, D. Loukas, G. Paspalaki, M. Diamantopoulou, G. Karathanasis, P. Kontaxakis, A. Panagiotou, I. Papavergou, N. Saoulidou, K. Theofilatos, K. Vellidis, G. Bakas, K. Kousouris, I. Papakrivopoulos, G. Tsipolitis, I. Evangelou, C. Foudas, P. Gianneios, P. Katsoulis, P. Kokkas, S. Mallios, K. Manitara, N. Manthos, I. Papadopoulos, E. Paradas, J. Strologas, F. A. Triantis, D. Tsitsonis, M. Bartók, M. Csanad, P. Major, K. Mandal, A. Mehta, M. I. Nagy, G. Pasztor, O. Surányi, G. I. Veres, G. Bencze, C. Hajdu, D. Horvath, Ã. Hunyadi, F. Sikler, T. Ã. Vámi, V. Veszpremi, G. Vesztergombi, N. Beni, S. Czellar, J. Karancsi, A. Makovec, J. Molnar, Z. Szillasi, P. Raics, D. Teyssier, Z. L. Trocsanyi, B. Ujvari, T. F. Csorgo, F. Nemes, T. Novak, S. Choudhury, J. R. Komaragiri, P. C. Tiwari, S. Bahinipati, C. Kar, G. Kole, P. Mal, V. K. Muraleedharan Nair Bindhu, A. Nayak, S. Roy Chowdhury, D. K. Sahoo, S. K. Swain, S. Bansal, S. B. Beri, V. Bhatnagar, S. Chauhan, R. Chawla, N. Dhingra, R. Gupta, A. Kaur, M. Kaur, S. Kaur, P. Kumari, M. Lohan, M. Meena, K. Sandeep, S. Sharma, J. B. Singh, A. K. Virdi, G. Walia, A. Bhardwaj, B. C. Choudhary, R. B. Garg, M. Gola, S. Keshri, Ashok Kumar, S. Malhotra, M. Naimuddin, P. Priyanka, K. Ranjan, Aashaq Shah, R. Sharma, R. Bhardwaj, M. Bharti, R. Bhattacharya, S. Bhattacharya, U. Bhawandeep, D. Bhowmik, S. Dey, S. Dutta, S. Ghosh, M. Maity, K. Mondal, S. Nandan, A. Purohit, P. K. Rout, A. Roy, G. Saha, S. Sarkar, T. Sarkar, M. Sharan, B. Singh, S. Thakur, P. K. Behera, A. Muhammad, R. Chudasama, D. Dutta, V. Jha, V. Kumar, D. K. Mishra, P. K. Netrakanti, L. M. Pant, P. Shukla, T. Aziz, M. A. Bhat, S. Dugad, G. B. Mohanty, N. Sur, RavindraKumar Verma, S. Banerjee, S. Bhattacharya, S. Chatterjee, P. Das, M. Guchait, S. Karmakar, S. Kumar, G. Majumder, K. Mazumdar, N. Sahoo, S. Sawant, S. Chauhan, S. Dube, V. Hegde, A. Kapoor, K. Kothekar, S. Pandey, A. Rane, A. Rastogi, S. Sharma, S. Chenarani, E. Eskandari Tadavani, S. M. Etesami, M. Khakzad, M. Mohammadi Najafabadi, M. Naseri, F. Rezaei Hosseinabadi, B. Safarzadeh, M. Felcini, M. Grunewald, M. Abbrescia, C. Calabria, A. Colaleo, D. Creanza, L. Cristella, N. De Filippis, M. De Palma, A. Di Florio, L. Fiore, A. Gelmi, G. Iaselli, M. Ince, S. Lezki, G. Maggi, M. Maggi, G. Miniello, S. My, S. Nuzzo, A. Pompili, G. Pugliese, R. Radogna, A. Ranieri, G. Selvaggi, L. Silvestris, R. Venditti, P. Verwilligen, G. Abbiendi, C. Battilana, D. Bonacorsi, L. Borgonovi, S. Braibant-Giacomelli, R. Campanini, P. Capiluppi, A. Castro, F. R. Cavallo, C. Ciocca, G. Codispoti, M. Cuffiani, G. M. Dallavalle, F. Fabbri, A. Fanfani, E. Fontanesi, P. Giacomelli, C. Grandi, L. Guiducci, F. Iemmi, S. Lo Meo, S. Marcellini, G. Masetti, F. L. Navarria, A. Perrotta, F. Primavera, A. M. Rossi, T. Rovelli, G. P. Siroli, N. Tosi, S. Albergo, S. Costa, A. Di Mattia, R. Potenza, A. Tricomi, C. Tuve, G. Barbagli, R. Ceccarelli, K. Chatterjee, V. Ciulli, C. Civinini, R. D’Alessandro, E. Focardi, G. Latino, P. Lenzi, M. Meschini, S. Paoletti, L. Russo, G. Sguazzoni, D. Strom, L. Viliani, L. Benussi, S. Bianco, F. Fabbri, D. Piccolo, M. Bozzo, F. Ferro, R. Mulargia, E. Robutti, S. Tosi, A. Benaglia, A. Beschi, F. Brivio, V. Ciriolo, S. Di Guida, M. E. Dinardo, P. Dini, S. Fiorendi, S. Gennai, A. Ghezzi, P. Govoni, M. Malberti, S. Malvezzi, D. Menasce, F. Monti, L. Moroni, G. Ortona, M. Paganoni, D. Pedrini, S. Ragazzi, T. Tabarelli de Fatis, D. Zuolo, S. Buontempo, N. Cavallo, A. De Iorio, A. Di Crescenzo, F. Fabozzi, F. Fienga, G. Galati, A. O. M. Iorio, L. Lista, S. Meola, P. Paolucci, B. Rossi, C. Sciacca, E. Voevodina, P. Azzi, N. Bacchetta, D. Bisello, A. Boletti, A. Bragagnolo, R. Carlin, P. Checchia, M. Dall’Osso, P. De Castro Manzano, T. Dorigo, U. Dosselli, F. Gasparini, U. Gasparini, A. Gozzelino, S. Y. Hoh, P. Lujan, M. Margoni, A. T. Meneguzzo, J. Pazzini, M. Presilla, P. Ronchese, R. Rossin, F. Simonetto, A. Tiko, E. Torassa, M. Tosi, M. Zanetti, P. Zotto, G. Zumerle, A. Braghieri, P. Montagna, S. P. Ratti, V. Re, M. Ressegotti, C. Riccardi, P. Salvini, I. Vai, P. Vitulo, M. Biasini, G. M. Bilei, C. Cecchi, D. Ciangottini, L. Fanò, P. Lariccia, R. Leonardi, E. Manoni, G. Mantovani, V. Mariani, M. Menichelli, A. Rossi, A. Santocchia, D. Spiga, K. Androsov, P. Azzurri, G. Bagliesi, V. Bertacchi, L. Bianchini, T. Boccali, R. Castaldi, M. A. Ciocci, R. Dell’Orso, G. Fedi, F. Fiori, L. Giannini, A. Giassi, M. T. Grippo, F. Ligabue, E. Manca, G. Mandorli, A. Messineo, F. Palla, A. Rizzi, G. Rolandi, A. Scribano, P. Spagnolo, R. Tenchini, G. Tonelli, N. Turini, A. Venturi, P. G. Verdini, F. Cavallari, M. Cipriani, D. Del Re, E. Di Marco, M. Diemoz, S. Gelli, E. Longo, B. Marzocchi, P. Meridiani, G. Organtini, F. Pandolfi, R. Paramatti, F. Preiato, C. Quaranta, S. Rahatlou, C. Rovelli, F. Santanastasio, L. Soffi, N. Amapane, R. Arcidiacono, S. Argiro, M. Arneodo, N. Bartosik, R. Bellan, C. Biino, A. Cappati, N. Cartiglia, F. Cenna, S. Cometti, M. Costa, R. Covarelli, N. Demaria, B. Kiani, C. Mariotti, S. Maselli, E. Migliore, V. Monaco, E. Monteil, M. Monteno, M. M. Obertino, L. Pacher, N. Pastrone, M. Pelliccioni, G. L. Pinna Angioni, A. Romero, M. Ruspa, R. Sacchi, R. Salvatico, K. Shchelina, V. Sola, A. Solano, D. Soldi, A. Staiano, S. Belforte, V. Candelise, M. Casarsa, F. Cossutti, A. Da Rold, G. Della Ricca, F. Vazzoler, A. Zanetti, B. Kim, D. H. Kim, G. N. Kim, M. S. Kim, J. Lee, S. W. Lee, C. S. Moon, Y. D. Oh, S. I. Pak, S. Sekmen, D. C. Son, Y. C. Yang, H. Kim, D. H. Moon, G. Oh, B. Francois, T. J. Kim, J. Park, S. Cho, S. Choi, Y. Go, D. Gyun, S. Ha, B. Hong, Y. Jo, K. Lee, K. S. Lee, S. Lee, J. Lim, J. Park, S. K. Park, Y. Roh, J. Goh, H. S. Kim, J. Almond, J. H. Bhyun, J. Choi, S. Jeon, J. Kim, J. S. Kim, H. Lee, K. Lee, S. Lee, K. Nam, S. B. Oh, B. C. Radburn-Smith, S. h. Seo, U. K. Yang, H. D. Yoo, I. Yoon, G. B. Yu, D. Jeon, H. Kim, J. H. Kim, J. S. H. Lee, I. C. Park, Y. Choi, C. Hwang, Y. Jeong, J. Lee, Y. Lee, I. Yu, V. Veckalns, V. Dudenas, A. Juodagalvis, J. Vaitkus, Z. A. Ibrahim, F. Mohamad Idris, W. A. T. Wan Abdullah, M. N. Yusli, Z. Zolkapli, J. F. Benitez, A. Castaneda Hernandez, J. A. Murillo Quijada, L. Valencia Palomo, H. Castilla-Valdez, E. De La Cruz-Burelo, M. C. Duran-Osuna, I. Heredia-De La Cruz, R. Lopez-Fernandez, R. I. Rabadan-Trejo, G. Ramirez-Sanchez, R. Reyes-Almanza, A. Sanchez-Hernandez, S. Carrillo Moreno, C. Oropeza Barrera, M. Ramirez-Garcia, F. Vazquez Valencia, J. Eysermans, I. Pedraza, H. A. Salazar Ibarguen, C. Uribe Estrada, A. Morelos Pineda, N. Raicevic, D. Krofcheck, S. Bheesette, P. H. Butler, A. Ahmad, M. Ahmad, Q. Hassan, H. R. Hoorani, W. A. Khan, M. A. Shah, M. Shoaib, M. Waqas, V. Avati, L. Grzanka, M. Malawski, H. Bialkowska, M. Bluj, B. Boimska, M. Górski, M. Kazana, M. Szleper, P. Zalewski, K. Bunkowski, A. Byszuk, K. Doroba, A. Kalinowski, M. Konecki, J. Krolikowski, M. Misiura, M. Olszewski, A. Pyskir, M. Walczak, M. Araujo, P. Bargassa, D. Bastos, A. Di Francesco, P. Faccioli, B. Galinhas, M. Gallinaro, J. Hollar, N. Leonardo, J. Seixas, G. Strong, O. Toldaiev, J. Varela, V. Alexakhin, P. Bunin, Y. Ershov, M. Gavrilenko, I. Golutvin, A. Kamenev, V. Karjavine, I. Kashunin, V. Korenkov, G. Kozlov, A. Lanev, A. Malakhov, V. Matveev, P. Moisenz, V. Palichik, V. Perelygin, S. Shmatov, S. Shulha, B. S. Yuldashev, A. Zarubin, L. Chtchipounov, V. Golovtsov, Y. Ivanov, V. Kim, E. Kuznetsova, P. Levchenko, V. Murzin, V. Oreshkin, I. Smirnov, D. Sosnov, V. Sulimov, L. Uvarov, A. Vorobyev, Yu. Andreev, A. Dermenev, S. Gninenko, N. Golubev, A. Karneyeu, M. Kirsanov, N. Krasnikov, A. Pashenkov, D. Tlisov, A. Toropin, V. Epshteyn, V. Gavrilov, N. Lychkovskaya, A. Nikitenko, V. Popov, I. Pozdnyakov, G. Safronov, A. Spiridonov, A. Stepennov, V. Stolin, M. Toms, E. Vlasov, A. Zhokin, T. Aushev, M. Chadeeva, D. Philippov, E. Popova, V. Rusinov, V. Andreev, M. Azarkin, I. Dremin, M. Kirakosyan, A. Terkulov, A. Belyaev, E. Boos, A. Ershov, A. Gribushin, L. Khein, V. Klyukhin, O. Kodolova, I. Lokhtin, O. Lukina, S. Obraztsov, S. Petrushanko, V. Savrin, A. Snigirev, A. Barnyakov, V. Blinov, T. Dimova, L. Kardapoltsev, Y. Skovpen, I. Azhgirey, I. Bayshev, S. Bitioukov, V. Kachanov, D. Konstantinov, P. Mandrik, V. Petrov, R. Ryutin, S. Slabospitskii, A. Sobol, S. Troshin, N. Tyurin, A. Uzunian, A. Volkov, A. Babaev, A. Iuzhakov, V. Okhotnikov, V. Borchsh, V. Ivantchenko, E. Tcherniaev, P. Adzic, P. Cirkovic, D. Devetak, M. Dordevic, P. Milenovic, J. Milosevic, M. Stojanovic, M. Aguilar-Benitez, J. Alcaraz Maestre, A. Ãlvarez Fernández, I. Bachiller, M. Barrio Luna, J. A. Brochero Cifuentes, C. A. Carrillo Montoya, M. Cepeda, M. Cerrada, N. Colino, B. De La Cruz, A. Delgado Peris, C. Fernandez Bedoya, J. P. Fernández Ramos, J. Flix, M. C. Fouz, O. Gonzalez Lopez, S. Goy Lopez, J. M. Hernandez, M. I. Josa, D. Moran, Ã. Navarro Tobar, A. Pérez-Calero Yzquierdo, J. Puerta Pelayo, I. Redondo, L. Romero, S. Sánchez Navas, M. S. Soares, A. Triossi, C. Willmott, C. Albajar, J. F. de Trocóniz, J. Cuevas, C. Erice, J. Fernandez Menendez, S. Folgueras, I. Gonzalez Caballero, J. R. González Fernández, E. Palencia Cortezon, V. Rodríguez Bouza, S. Sanchez Cruz, J. M. Vizan Garcia, I. J. Cabrillo, A. Calderon, B. Chazin Quero, J. Duarte Campderros, M. Fernandez, P. J. Fernández Manteca, A. García Alonso, G. Gomez, C. Martinez Rivero, P. Martinez Ruiz del Arbol, F. Matorras, J. Piedra Gomez, C. Prieels, T. Rodrigo, A. Ruiz-Jimeno, L. Scodellaro, N. Trevisani, I. Vila, K. Malagalage, W. G. D. Dharmaratna, N. Wickramage, D. Abbaneo, B. Akgun, E. Auffray, G. Auzinger, J. Baechler, P. Baillon, A. H. Ball, D. Barney, J. Bendavid, M. Bianco, A. Bocci, E. Bossini, C. Botta, E. Brondolin, T. Camporesi, A. Caratelli, G. Cerminara, E. Chapon, G. Cucciati, D. d’Enterria, A. Dabrowski, N. Daci, V. Daponte, A. David, A. De Roeck, N. Deelen, M. Deile, M. Dobson, M. Dünser, N. Dupont, A. Elliott-Peisert, F. Fallavollita, D. Fasanella, G. Franzoni, J. Fulcher, W. Funk, S. Giani, D. Gigi, A. Gilbert, K. Gill, F. Glege, M. Gruchala, M. Guilbaud, D. Gulhan, J. Hegeman, C. Heidegger, Y. Iiyama, V. Innocente, A. Jafari, P. Janot, O. Karacheban, J. Kaspar, J. Kieseler, M. Krammer, C. Lange, P. Lecoq, C. Lourenço, L. Malgeri, M. Mannelli, A. Massironi, F. Meijers, J. A. Merlin, S. Mersi, E. Meschi, F. Moortgat, M. Mulders, J. Ngadiuba, S. Nourbakhsh, S. Orfanelli, L. Orsini, F. Pantaleo, L. Pape, E. Perez, M. Peruzzi, A. Petrilli, G. Petrucciani, A. Pfeiffer, M. Pierini, F. M. Pitters, M. Quinto, D. Rabady, A. Racz, M. Rovere, H. Sakulin, C. Schäfer, C. Schwick, M. Selvaggi, A. Sharma, P. Silva, W. Snoeys, P. Sphicas, A. Stakia, J. Steggemann, V. R. Tavolaro, D. Treille, A. Tsirou, A. Vartak, M. Verzetti, W. D. Zeuner, L. Caminada, K. Deiters, W. Erdmann, R. Horisberger, Q. Ingram, H. C. Kaestli, D. Kotlinski, U. Langenegger, T. Rohe, S. A. Wiederkehr, M. Backhaus, P. Berger, N. Chernyavskaya, G. Dissertori, M. Dittmar, M. Donegà, C. Dorfer, T. A. Gómez Espinosa, C. Grab, D. Hits, T. Klijnsma, W. Lustermann, R. A. Manzoni, M. Marionneau, M. T. Meinhard, F. Micheli, P. Musella, F. Nessi-Tedaldi, F. Pauss, G. Perrin, L. Perrozzi, S. Pigazzini, M. Reichmann, C. Reissel, T. Reitenspiess, D. Ruini, D. A. Sanz Becerra, M. Schönenberger, L. Shchutska, M. L. Vesterbacka Olsson, R. Wallny, D. H. Zhu, T. K. Aarrestad, C. Amsler, D. Brzhechko, M. F. Canelli, A. De Cosa, R. Del Burgo, S. Donato, C. Galloni, B. Kilminster, S. Leontsinis, V. M. Mikuni, I. Neutelings, G. Rauco, P. Robmann, D. Salerno, K. Schweiger, C. Seitz, Y. Takahashi, S. Wertz, A. Zucchetta, T. H. Doan, C. M. Kuo, W. Lin, S. S. Yu, P. Chang, Y. Chao, K. F. Chen, P. H. Chen, W.-S. Hou, Y. y. Li, R.-S. Lu, E. Paganis, A. Psallidas, A. Steen, B. Asavapibhop, N. Srimanobhas, N. Suwonjandee, A. Bat, F. Boran, S. Cerci, S. Damarseckin, Z. S. Demiroglu, F. Dolek, C. Dozen, I. Dumanoglu, G. Gokbulut, EmineGurpinar Guler, Y. Guler, I. Hos, C. Isik, E. E. Kangal, O. Kara, A. Kayis Topaksu, U. Kiminsu, M. Oglakci, G. Onengut, K. Ozdemir, S. Ozturk, A. E. Simsek, D. Sunar Cerci, U. G. Tok, S. Turkcapar, I. S. Zorbakir, C. Zorbilmez, B. Isildak, G. Karapinar, M. Yalvac, I. O. Atakisi, E. Gülmez, M. Kaya, O. Kaya, B. Kaynak, Ö. Özçelik, S. Ozkorucuklu, S. Tekten, S. Tekten, E. A. Yetkin, A. Cakir, Y. Komurcu, S. Sen, B. Grynyov, L. Levchuk, F. Ball, E. Bhal, S. Bologna, J. J. Brooke, D. Burns, E. Clement, D. Cussans, O. Davignon, H. Flacher, J. Goldstein, G. P. Heath, H. F. Heath, L. Kreczko, S. Paramesvaran, B. Penning, T. Sakuma, S. Seif El Nasr-Storey, D. Smith, V. J. Smith, J. Taylor, A. Titterton, K. W. Bell, A. Belyaev, C. Brew, R. M. Brown, D. Cieri, D. J. A. Cockerill, J. A. Coughlan, K. Harder, S. Harper, J. Linacre, K. Manolopoulos, D. M. Newbold, E. Olaiya, D. Petyt, T. Reis, T. Schuh, C. H. Shepherd-Themistocleous, A. Thea, I. R. Tomalin, T. Williams, W. J. Womersley, R. Bainbridge, P. Bloch, J. Borg, S. Breeze, O. Buchmuller, A. Bundock, GurpreetSingh CHAHAL, D. Colling, P. Dauncey, G. Davies, M. Della Negra, R. Di Maria, P. Everaerts, G. Hall, G. Iles, T. James, M. Komm, C. Laner, L. Lyons, A.-M. Magnan, S. Malik, A. Martelli, V. Milosevic, J. Nash, V. Palladino, M. Pesaresi, D. M. Raymond, A. Richards, A. Rose, E. Scott, C. Seez, A. Shtipliyski, M. Stoye, T. Strebler, S. Summers, A. Tapper, K. Uchida, T. Virdee, N. Wardle, D. Winterbottom, J. Wright, A. G. Zecchinelli, S. C. Zenz, J. E. Cole, P. R. Hobson, A. Khan, P. Kyberd, C. K. Mackay, A. Morton, I. D. Reid, L. Teodorescu, S. Zahid, K. Call, J. Dittmann, K. Hatakeyama, C. Madrid, B. Mcmaster, N. Pastika, C. Smith, R. Bartek, A. Dominguez, R. Uniyal, A. Buccilli, S. I. Cooper, C. Henderson, P. Rumerio, C. West, D. Arcaro, T. Bose, Z. Demiragli, D. Gastler, S. Girgis, D. Pinna, C. Richardson, J. Rohlf, D. Sperka, I. Suarez, L. Sulak, D. Zou, G. Benelli, B. Burkle, X. Coubez, D. Cutts, M. Hadley, J. Hakala, U. Heintz, J. M. Hogan, K. H. M. Kwok, E. Laird, G. Landsberg, J. Lee, Z. Mao, M. Narain, S. Sagir, R. Syarif, E. Usai, D. Yu, R. Band, C. Brainerd, R. Breedon, M. Calderon De La Barca Sanchez, M. Chertok, J. Conway, R. Conway, P. T. Cox, R. Erbacher, C. Flores, G. Funk, F. Jensen, W. Ko, O. Kukral, R. Lander, M. Mulhearn, D. Pellett, J. Pilot, M. Shi, D. Stolp, D. Taylor, K. Tos, M. Tripathi, Z. Wang, F. Zhang, M. Bachtis, C. Bravo, R. Cousins, A. Dasgupta, A. Florent, J. Hauser, M. Ignatenko, N. Mccoll, S. Regnard, D. Saltzberg, C. Schnaible, V. Valuev, K. Burt, R. Clare, J. W. Gary, S. M. A. Ghiasi Shirazi, G. Hanson, G. Karapostoli, E. Kennedy, O. R. Long, M. Olmedo Negrete, M. I. Paneva, W. Si, L. Wang, H. Wei, S. Wimpenny, B. R. Yates, Y. Zhang, J. G. Branson, P. Chang, S. Cittolin, M. Derdzinski, R. Gerosa, D. Gilbert, B. Hashemi, D. Klein, V. Krutelyov, J. Letts, M. Masciovecchio, S. May, S. Padhi, M. Pieri, V. Sharma, M. Tadel, F. Würthwein, A. Yagil, G. Zevi Della Porta, N. Amin, R. Bhandari, C. Campagnari, M. Citron, V. Dutta, M. Franco Sevilla, L. Gouskos, J. Incandela, B. Marsh, H. Mei, A. Ovcharova, H. Qu, J. Richman, U. Sarica, D. Stuart, S. Wang, J. Yoo, D. Anderson, A. Bornheim, J. M. Lawhorn, N. Lu, H. B. Newman, T. Q. Nguyen, J. Pata, M. Spiropulu, J. R. Vlimant, S. Xie, Z. Zhang, R. Y. Zhu, M. B. Andrews, T. Ferguson, T. Mudholkar, M. Paulini, M. Sun, I. Vorobiev, M. Weinberg, J. P. Cumalat, W. T. Ford, A. Johnson, E. MacDonald, T. Mulholland, R. Patel, A. Perloff, K. Stenson, K. A. Ulmer, S. R. Wagner, J. Alexander, J. Chaves, Y. Cheng, J. Chu, A. Datta, A. Frankenthal, K. Mcdermott, N. Mirman, J. R. Patterson, D. Quach, A. Rinkevicius, A. Ryd, S. M. Tan, Z. Tao, J. Thom, P. Wittich, M. Zientek, S. Abdullin, M. Albrow, M. Alyari, G. Apollinari, A. Apresyan, A. Apyan, S. Banerjee, L. A. T. Bauerdick, A. Beretvas, J. Berryhill, P. C. Bhat, K. Burkett, J. N. Butler, A. Canepa, G. B. Cerati, H. W. K. Cheung, F. Chlebana, M. Cremonesi, J. Duarte, V. D. Elvira, J. Freeman, Z. Gecse, E. Gottschalk, L. Gray, D. Green, S. Grünendahl, O. Gutsche, Allison-Reinsvold Hall, J. Hanlon, R. M. Harris, S. Hasegawa, R. Heller, J. Hirschauer, Z. Hu, B. Jayatilaka, S. Jindariani, M. Johnson, U. Joshi, B. Klima, M. J. Kortelainen, B. Kreis, S. Lammel, J. Lewis, D. Lincoln, R. Lipton, M. Liu, T. Liu, J. Lykken, K. Maeshima, J. M. Marraffino, D. Mason, P. McBride, P. Merkel, S. Mrenna, S. Nahn, V. O’Dell, V. Papadimitriou, K. Pedro, C. Pena, G. Rakness, F. Ravera, L. Ristori, B. Schneider, E. Sexton-Kennedy, N. Smith, A. Soha, W. J. Spalding, L. Spiegel, S. Stoynev, J. Strait, N. Strobbe, L. Taylor, S. Tkaczyk, N. V. Tran, L. Uplegger, E. W. Vaandering, C. Vernieri, M. Verzocchi, R. Vidal, M. Wang, H. A. Weber, D. Acosta, P. Avery, P. Bortignon, D. Bourilkov, A. Brinkerhoff, L. Cadamuro, A. Carnes, V. Cherepanov, D. Curry, F. Errico, R. D. Field, S. V. Gleyzer, B. M. Joshi, M. Kim, J. Konigsberg, A. Korytov, K. H. Lo, P. Ma, K. Matchev, N. Menendez, G. Mitselmakher, D. Rosenzweig, K. Shi, J. Wang, S. Wang, X. Zuo, Y. R. Joshi, S. Linn, T. Adams, A. Askew, S. Hagopian, V. Hagopian, K. F. Johnson, R. Khurana, T. Kolberg, G. Martinez, T. Perry, H. Prosper, C. Schiber, R. Yohay, M. M. Baarmand, V. Bhopatkar, M. Hohlmann, D. Noonan, M. Rahmani, M. Saunders, F. Yumiceva, M. R. Adams, L. Apanasevich, D. Berry, R. R. Betts, R. Cavanaugh, X. Chen, S. Dittmer, O. Evdokimov, C. E. Gerber, D. A. Hangal, D. J. Hofman, K. Jung, C. Mills, T. Roy, M. B. Tonjes, N. Varelas, H. Wang, X. Wang, Z. Wu, J. Zhang, M. Alhusseini, B. Bilki, W. Clarida, K. Dilsiz, S. Durgut, R. P. Gandrajula, M. Haytmyradov, V. Khristenko, O. K. Köseyan, J.-P. Merlo, A. Mestvirishvili, A. Moeller, J. Nachtman, H. Ogul, Y. Onel, F. Ozok, A. Penzo, C. Snyder, E. Tiras, J. Wetzel, B. Blumenfeld, A. Cocoros, N. Eminizer, D. Fehling, L. Feng, A. V. Gritsan, W. T. Hung, P. Maksimovic, J. Roskes, M. Swartz, M. Xiao, C. Baldenegro Barrera, P. Baringer, A. Bean, S. Boren, J. Bowen, A. Bylinkin, T. Isidori, S. Khalil, J. King, A. Kropivnitskaya, D. Majumder, W. Mcbrayer, N. Minafra, M. Murray, C. Rogan, C. Royon, S. Sanders, E. Schmitz, J. D. Tapia Takaki, Q. Wang, J. Williams, S. Duric, A. Ivanov, K. Kaadze, D. Kim, Y. Maravin, D. R. Mendis, T. Mitchell, A. Modak, A. Mohammadi, F. Rebassoo, D. Wright, A. Baden, O. Baron, A. Belloni, S. C. Eno, Y. Feng, C. Ferraioli, N. J. Hadley, S. Jabeen, G. Y. Jeng, R. G. Kellogg, J. Kunkle, A. C. Mignerey, S. Nabili, F. Ricci-Tam, M. Seidel, Y. H. Shin, A. Skuja, S. C. Tonwar, K. Wong, D. Abercrombie, B. Allen, A. Baty, R. Bi, S. Brandt, W. Busza, I. A. Cali, M. D’Alfonso, G. Gomez Ceballos, M. Goncharov, P. Harris, D. Hsu, M. Hu, M. Klute, D. Kovalskyi, Y.-J. Lee, P. D. Luckey, B. Maier, A. C. Marini, C. Mcginn, C. Mironov, S. Narayanan, X. Niu, C. Paus, D. Rankin, C. Roland, G. Roland, Z. Shi, G. S. F. Stephans, K. Sumorok, K. Tatar, D. Velicanu, J. Wang, T. W. Wang, B. Wyslouch, A. C. Benvenuti, R. M. Chatterjee, A. Evans, P. Hansen, J. Hiltbrand, S. Kalafut, Y. Kubota, Z. Lesko, J. Mans, R. Rusack, M. A. Wadud, J. G. Acosta, S. Oliveros, E. Avdeeva, K. Bloom, D. R. Claes, C. Fangmeier, L. Finco, F. Golf, R. Gonzalez Suarez, R. Kamalieddin, I. Kravchenko, J. E. Siado, G. R. Snow, B. Stieger, A. Godshalk, C. Harrington, I. Iashvili, A. Kharchilava, C. Mclean, D. Nguyen, A. Parker, S. Rappoccio, B. Roozbahani, G. Alverson, E. Barberis, C. Freer, Y. Haddad, A. Hortiangtham, G. Madigan, D. M. Morse, T. Orimoto, L. Skinnari, A. Tishelman-Charny, T. Wamorkar, B. Wang, A. Wisecarver, D. Wood, S. Bhattacharya, J. Bueghly, T. Gunter, K. A. Hahn, N. Odell, M. H. Schmitt, K. Sung, M. Trovato, M. Velasco, R. Bucci, N. Dev, M. Hildreth, K. Hurtado Anampa, C. Jessop, D. J. Karmgard, K. Lannon, W. Li, N. Loukas, N. Marinelli, I. Mcalister, F. Meng, C. Mueller, Y. Musienko, M. Planer, R. Ruchti, P. Siddireddy, G. Smith, S. Taroni, M. Wayne, A. Wightman, M. Wolf, A. Woodard, J. Alimena, B. Bylsma, L. S. Durkin, S. Flowers, B. Francis, C. Hill, W. Ji, A. Lefeld, T. Y. Ling, B. L. Winer, S. Cooperstein, G. Dezoort, P. Elmer, J. Hardenbrook, N. Haubrich, S. Higginbotham, A. Kalogeropoulos, S. Kwan, D. Lange, M. T. Lucchini, J. Luo, D. Marlow, K. Mei, I. Ojalvo, J. Olsen, C. Palmer, P. Piroué, J. Salfeld-Nebgen, D. Stickland, C. Tully, Z. Wang, S. Malik, S. Norberg, A. Barker, V. E. Barnes, S. Das, L. Gutay, M. Jones, A. W. Jung, A. Khatiwada, B. Mahakud, D. H. Miller, G. Negro, N. Neumeister, C. C. Peng, S. Piperov, H. Qiu, J. F. Schulte, J. Sun, F. Wang, R. Xiao, W. Xie, T. Cheng, J. Dolen, N. Parashar, K. M. Ecklund, S. Freed, F. J. M. Geurts, M. Kilpatrick, Arun Kumar, W. Li, B. P. Padley, R. Redjimi, J. Roberts, J. Rorie, W. Shi, A. G. Stahl Leiton, Z. Tu, A. Zhang, A. Bodek, P. de Barbaro, R. Demina, Y. t. Duh, J. L. Dulemba, C. Fallon, T. Ferbel, M. Galanti, A. Garcia-Bellido, J. Han, O. Hindrichs, A. Khukhunaishvili, E. Ranken, P. Tan, R. Taus, B. Chiarito, J. P. Chou, Y. Gershtein, E. Halkiadakis, A. Hart, M. Heindl, E. Hughes, S. Kaplan, S. Kyriacou, I. Laflotte, A. Lath, R. Montalvo, K. Nash, M. Osherson, H. Saka, S. Salur, S. Schnetzer, D. Sheffield, S. Somalwar, R. Stone, S. Thomas, P. Thomassen, H. Acharya, A. G. Delannoy, J. Heideman, G. Riley, S. Spanier, O. Bouhali, A. Celik, M. Dalchenko, M. De Mattia, A. Delgado, S. Dildick, R. Eusebi, J. Gilmore, T. Huang, T. Kamon, S. Luo, D. Marley, R. Mueller, D. Overton, L. Perniè, D. Rathjens, A. Safonov, N. Akchurin, J. Damgov, F. De Guio, S. Kunori, K. Lamichhane, S. W. Lee, T. Mengke, S. Muthumuni, T. Peltola, S. Undleeb, I. Volobouev, Z. Wang, A. Whitbeck, S. Greene, A. Gurrola, R. Janjam, W. Johns, C. Maguire, A. Melo, H. Ni, K. Padeken, F. Romeo, P. Sheldon, S. Tuo, J. Velkovska, M. Verweij, M. W. Arenton, P. Barria, B. Cox, G. Cummings, R. Hirosky, M. Joyce, A. Ledovskoy, C. Neu, B. Tannenwald, Y. Wang, E. Wolfe, F. Xia, R. Harr, P. E. Karchin, N. Poudyal, J. Sturdy, P. Thapa, S. Zaleski, J. Buchanan, C. Caillol, D. Carlsmith, S. Dasu, I. De Bruyn, L. Dodd, B. Gomber, M. Grothe, M. Herndon, A. Hervé, U. Hussain, P. Klabbers, A. Lanaro, K. Long, R. Loveless, T. Ruggles, A. Savin, V. Sharma, W. H. Smith, N. Woods, CMS Collaboration

**Affiliations:** 1 Yerevan Physics Institute, Yerevan, Armenia; 2 Institut für Hochenergiephysik, Wien, Austria; 3 Institute for Nuclear Problems, Minsk, Belarus; 4 Universiteit Antwerpen, Antwerpen, Belgium; 5 Vrije Universiteit Brussel, Brussel, Belgium; 6 Université Libre de Bruxelles, Bruxelles, Belgium; 7 Ghent University, Ghent, Belgium; 8 Université Catholique de Louvain, Louvain-la-Neuve, Belgium; 9 Centro Brasileiro de Pesquisas Fisicas, Rio de Janeiro, Brazil; 10 Universidade do Estado do Rio de Janeiro, Rio de Janeiro, Brazil; 11 Universidade Estadual Paulista, Universidade Federal do ABC, São Paulo, Brazil; 12 Institute for Nuclear Research and Nuclear Energy, Bulgarian Academy of Sciences, Sofia, Bulgaria; 13 University of Sofia, Sofia, Bulgaria; 14 Beihang University, Beijing, China; 15 Institute of High Energy Physics, Beijing, China; 16 State Key Laboratory of Nuclear Physics and Technology, Peking University, Beijing, China; 17 Tsinghua University, Beijing, China; 18 Universidad de Los Andes, Bogota, Colombia; 19 Universidad de Antioquia, Medellin, Colombia; 20 University of Split, Faculty of Electrical Engineering, Mechanical Engineering and Naval Architecture, Split, Croatia; 21 University of Split, Faculty of Science, Split, Croatia; 22 Institute Rudjer Boskovic, Zagreb, Croatia; 23 University of Cyprus, Nicosia, Cyprus; 24 Charles University, Prague, Czech Republic; 25 Escuela Politecnica Nacional, Quito, Ecuador; 26 Universidad San Francisco de Quito, Quito, Ecuador; 27 Academy of Scientific Research and Technology of the Arab Republic of Egypt, Egyptian Network of High Energy Physics, Cairo, Egypt; 28 National Institute of Chemical Physics and Biophysics, Tallinn, Estonia; 29 Department of Physics, University of Helsinki, Helsinki, Finland; 30 Helsinki Institute of Physics, Helsinki, Finland; 31 Lappeenranta University of Technology, Lappeenranta, Finland; 32 IRFU, CEA, Université Paris-Saclay, Gif-sur-Yvette, France; 33 Laboratoire Leprince-Ringuet, Ecole polytechnique, CNRS/IN2P3, Université Paris-Saclay, Palaiseau, France; 34 Université de Strasbourg, CNRS, IPHC UMR 7178, Strasbourg, France; 35 Centre de Calcul de l’Institut National de Physique Nucleaire et de Physique des Particules, CNRS/IN2P3, Villeurbanne, France; 36 Université de Lyon, Université Claude Bernard Lyon 1, CNRS-IN2P3, Institut de Physique Nucléaire de Lyon, Villeurbanne, France; 37 Georgian Technical University, Tbilisi, Georgia; 38 Tbilisi State University, Tbilisi, Georgia; 39 RWTH Aachen University, I. Physikalisches Institut, Aachen, Germany; 40 RWTH Aachen University, III. Physikalisches Institut A, Aachen, Germany; 41 RWTH Aachen University, III. Physikalisches Institut B, Aachen, Germany; 42 Deutsches Elektronen-Synchrotron, Hamburg, Germany; 43 University of Hamburg, Hamburg, Germany; 44 Karlsruher Institut fuer Technologie, Karlsruhe, Germany; 45 Institute of Nuclear and Particle Physics (INPP), NCSR Demokritos, Aghia Paraskevi, Greece; 46 National and Kapodistrian University of Athens, Athens, Greece; 47 National Technical University of Athens, Athens, Greece; 48 University of Ioánnina, Ioánnina, Greece; 49 MTA-ELTE Lendület CMS Particle and Nuclear Physics Group, Eötvös Loránd University, Budapest, Hungary; 50 Wigner Research Centre for Physics, Budapest, Hungary; 51 Institute of Nuclear Research ATOMKI, Debrecen, Hungary; 52 Institute of Physics, University of Debrecen, Debrecen, Hungary; 53 Eszterhazy Karoly University, Karoly Robert Campus, Gyongyos, Hungary; 54 Indian Institute of Science (IISc), Bangalore, India; 55 National Institute of Science Education and Research, HBNI, Bhubaneswar, India; 56 Panjab University, Chandigarh, India; 57 University of Delhi, Delhi, India; 58 Saha Institute of Nuclear Physics, HBNI, Kolkata, India; 59 Indian Institute of Technology Madras, Madras, India; 60 Bhabha Atomic Research Centre, Mumbai, India; 61 Tata Institute of Fundamental Research-A, Mumbai, India; 62 Tata Institute of Fundamental Research-B, Mumbai, India; 63 Indian Institute of Science Education and Research (IISER), Pune, India; 64 Institute for Research in Fundamental Sciences (IPM), Tehran, Iran; 65 University College Dublin, Dublin, Ireland; 66 INFN Sezione di Bari, Università di Bari, Politecnico di Bari, Bari, Italy; 67 INFN Sezione di Bologna, Università di Bologna, Bologna, Italy; 68 INFN Sezione di Catania, Università di Catania, Catania, Italy; 69 INFN Sezione di Firenze, Università di Firenze, Firenze, Italy; 70 INFN Laboratori Nazionali di Frascati, Frascati, Italy; 71 INFN Sezione di Genova, Università di Genova, Genova, Italy; 72 INFN Sezione di Milano-Bicocca, Università di Milano-Bicocca, Milan, Italy; 73 INFN Sezione di Napoli, Università di Napoli ’Federico II’ , Napoli, Italy, Università della Basilicata, Potenza, Italy, Università G. Marconi, Rome, Italy; 74 INFN Sezione di Padova, Università di Padova, Padova, Italy, Università di Trento, Trento, Italy; 75 INFN Sezione di Pavia, Università di Pavia, Pavia, Italy; 76 INFN Sezione di Perugia, Università di Perugia, Perugia, Italy; 77 INFN Sezione di Pisa, Università di Pisa, Scuola Normale Superiore di Pisa, Pisa, Italy; 78 INFN Sezione di Roma, Sapienza Università di Roma, Rome, Italy; 79 INFN Sezione di Torino, Università di Torino, Torino, Italy, Università del Piemonte Orientale, Novara, Italy; 80 INFN Sezione di Trieste, Università di Trieste, Trieste, Italy; 81 Kyungpook National University, Daegu, South Korea; 82 Chonnam National University, Institute for Universe and Elementary Particles, Kwangju, South Korea; 83 Hanyang University, Seoul, South Korea; 84 Korea University, Seoul, South Korea; 85 Department of Physics, Kyung Hee University, Seoul, South Korea; 86 Sejong University, Seoul, South Korea; 87 Seoul National University, Seoul, South Korea; 88 University of Seoul, Seoul, South Korea; 89 Sungkyunkwan University, Suwon, South Korea; 90 Riga Technical University, Riga, Latvia; 91 Vilnius University, Vilnius, Lithuania; 92 National Centre for Particle Physics, Universiti Malaya, Kuala Lumpur, Malaysia; 93 Universidad de Sonora (UNISON), Hermosillo, Mexico; 94 Centro de Investigacion y de Estudios Avanzados del IPN, Mexico City, Mexico; 95 Universidad Iberoamericana, Mexico City, Mexico; 96 Benemerita Universidad Autonoma de Puebla, Puebla, Mexico; 97 Universidad Autónoma de San Luis Potosí, San Luis Potosí, Mexico; 98 University of Montenegro, Podgorica, Montenegro; 99 University of Auckland, Auckland, New Zealand; 100 University of Canterbury, Christchurch, New Zealand; 101 National Centre for Physics, Quaid-I-Azam University, Islamabad, Pakistan; 102 AGH University of Science and Technology Faculty of Computer Science, Electronics and Telecommunications, Krakow, Poland; 103 National Centre for Nuclear Research, Swierk, Poland; 104 Institute of Experimental Physics, Faculty of Physics, University of Warsaw, Warsaw, Poland; 105 Laboratório de Instrumentação e Física Experimental de Partículas, Lisboa, Portugal; 106 Joint Institute for Nuclear Research, Dubna, Russia; 107 Petersburg Nuclear Physics Institute, Gatchina (St. Petersburg), Russia; 108 Institute for Nuclear Research, Moscow, Russia; 109 Institute for Theoretical and Experimental Physics named by A.I. Alikhanov of NRC ‘Kurchatov Institute’, Moscow, Russia; 110 Moscow Institute of Physics and Technology, Moscow, Russia; 111 National Research Nuclear University ’Moscow Engineering Physics Institute’ (MEPhI), Moscow, Russia; 112 P. N. Lebedev Physical Institute, Moscow, Russia; 113 Skobeltsyn Institute of Nuclear Physics, Lomonosov Moscow State University, Moscow, Russia; 114 Novosibirsk State University (NSU), Novosibirsk, Russia; 115 Institute for High Energy Physics of National Research Centre ‘Kurchatov Institute’, Protvino, Russia; 116 National Research Tomsk Polytechnic University, Tomsk, Russia; 117 Tomsk State University, Tomsk, Russia; 118 University of Belgrade: Faculty of Physics and VINCA Institute of Nuclear Sciences, Belgrade, Serbia; 119 Centro de Investigaciones Energéticas Medioambientales y Tecnológicas (CIEMAT), Madrid, Spain; 120 Universidad Autónoma de Madrid, Madrid, Spain; 121 Universidad de Oviedo, Instituto Universitario de Ciencias y Tecnologías Espaciales de Asturias (ICTEA), Oviedo, Spain; 122 Instituto de Física de Cantabria (IFCA), CSIC-Universidad de Cantabria, Santander, Spain; 123 University of Colombo, Colombo, Sri Lanka; 124 University of Ruhuna, Department of Physics, Matara, Sri Lanka; 125 CERN, European Organization for Nuclear Research, Geneva, Switzerland; 126 Paul Scherrer Institut, Villigen, Switzerland; 127 ETH Zurich-Institute for Particle Physics and Astrophysics (IPA), Zurich, Switzerland; 128 Universität Zürich, Zurich, Switzerland; 129 National Central University, Chung-Li, Taiwan; 130 National Taiwan University (NTU), Taipei, Taiwan; 131 Chulalongkorn University, Faculty of Science, Department of Physics, Bangkok, Thailand; 132 Ãukurova University, Physics Department, Science and Art Faculty, Adana, Turkey; 133 Physics Department, Middle East Technical University, Ankara, Turkey; 134 Bogazici University, Istanbul, Turkey; 135 Istanbul Technical University, Istanbul, Turkey; 136 Institute for Scintillation Materials of National Academy of Science of Ukraine, Kharkov, Ukraine; 137 National Scientific Center, Kharkov Institute of Physics and Technology, Kharkov, Ukraine; 138 University of Bristol, Bristol, UK; 139 Rutherford Appleton Laboratory, Didcot, UK; 140 Imperial College, London, UK; 141 Brunel University, Uxbridge, UK; 142 Baylor University, Waco, USA; 143 Catholic University of America, Washington DC, USA; 144 The University of Alabama, Tuscaloosa, USA; 145 Boston University, Boston, USA; 146 Brown University, Providence, USA; 147 University of California, Davis, Davis, USA; 148 University of California, Los Angeles, USA; 149 University of California, Riverside, Riverside, USA; 150 University of California, San Diego, La Jolla, USA; 151 Department of Physics, University of California, Santa Barbara, Santa Barbara, USA; 152 California Institute of Technology, Pasadena, USA; 153 Carnegie Mellon University, Pittsburgh, USA; 154 University of Colorado Boulder, Boulder, USA; 155 Cornell University, Ithaca, USA; 156 Fermi National Accelerator Laboratory, Batavia, USA; 157 University of Florida, Gainesville, USA; 158 Florida International University, Miami, USA; 159 Florida State University, Tallahassee, USA; 160 Florida Institute of Technology, Melbourne, USA; 161 University of Illinois at Chicago (UIC), Chicago, USA; 162 The University of Iowa, Iowa City, USA; 163 Johns Hopkins University, Baltimore, USA; 164 The University of Kansas, Lawrence, USA; 165 Kansas State University, Manhattan, USA; 166 Lawrence Livermore National Laboratory, Livermore, USA; 167 University of Maryland, College Park, USA; 168 Massachusetts Institute of Technology, Cambridge, USA; 169 University of Minnesota, Minneapolis, USA; 170 University of Mississippi, Oxford, USA; 171 University of Nebraska-Lincoln, Lincoln, USA; 172 State University of New York at Buffalo, Buffalo, USA; 173 Northeastern University, Boston, USA; 174 Northwestern University, Evanston, USA; 175 University of Notre Dame, Notre Dame, USA; 176 The Ohio State University, Columbus, USA; 177 Princeton University, Princeton, USA; 178 University of Puerto Rico, Mayaguez, USA; 179 Purdue University, West Lafayette, USA; 180 Purdue University Northwest, Hammond, USA; 181 Rice University, Houston, USA; 182 University of Rochester, Rochester, USA; 183 Rutgers, The State University of New Jersey, Piscataway, USA; 184 University of Tennessee, Knoxville, USA; 185 Texas A & M University, College Station, USA; 186 Texas Tech University, Lubbock, USA; 187 Vanderbilt University, Nashville, USA; 188 University of Virginia, Charlottesville, USA; 189 Wayne State University, Detroit, USA; 190 University of Wisconsin - Madison, Madison, WI USA; 191 CERN, 1211 Geneva 23, Switzerland

## Abstract

The average total energy as well as its hadronic and electromagnetic components are measured with the CMS detector at pseudorapidities $-6.6<\eta <-5.2$ in proton-proton collisions at a centre-of-mass energy $\sqrt{s}=13\,\text {TeV}$. The results are presented as a function of the charged particle multiplicity in the region $|\eta |<2$. This measurement is sensitive to correlations induced by the underlying event structure over a very wide pseudorapidity region. The predictions of Monte Carlo event generators commonly used in collider experiments and ultra-high energy cosmic ray physics are compared to the data. All generators considered overestimate the fraction of energy going into hadrons.

## Introduction

The description of inclusive hadron production in high energy hadron-hadron collisions remains subject to significant theoretical uncertainties. At TeV energies the dominant source of secondary particle production is the fragmentation of quarks and gluons in semihard scattering [1], referred to as minijet production. However, various processes that cannot be directly calculated from first principles in quantum chromodynamics (QCD) also contribute to particle production, i.e. multiparton interactions (MPIs), and fragmentation of the remnants. Together with initial- and final-state radiation these additional particle production mechanisms are typically referred to as the underlying event and are modelled phenomenologically in Monte Carlo (MC) event generators with parameters tuned using data [2–4]. In addition, especially in the forward phase space, diffractive processes play an important role [5]. Furthermore, final-state parton rescattering effects, a possible hydrodynamical phase transition, or other collective phenomena can impact and modify particle production in hadron-hadron collisions at high energies [6].

The energy carried by particles emitted into the very forward region ($-6.6<\eta <-5.2$) covered by the CASTOR calorimeter [7] of the CMS experiment was shown to be a powerful probe of the activity of the underlying event [8, 9]. For the first time measurements presented in this paper correlate the hadronic energy at very forward rapidities to the central region in proton-proton collisions, offering a new approach to the study of hadron production at the CERN LHC. Such measurements over a very large rapidity interval provide additional information on the underlying event compared to those based only on the central region, e.g. Refs. [10, 11].

The very forward region covered by the data contains the highest energy densities, $\mathrm{d}{}E$/$\mathrm{d}\eta$ [12, 13], so far observed in proton-proton collisions at the LHC. Therefore, the present results can improve event generators used in simulations of extensive air showers induced by cosmic rays at ultra-high energies [14]. Specifically, current air shower simulations are known to significantly underestimate muon production (see Ref. [15] and references therein). The fraction of the energy going into the production of electrons or photons rather than long-lived hadrons has a crucial impact on the muon production rate in extensive air showers, see Ref. [16]. Since CASTOR consists of separate electromagnetic and hadron calorimeters, the data presented here provide new information that may improve understanding of muon production in air showers.

## Experimental setup and Monte Carlo simulation

The main feature of the CMS apparatus is a superconducting solenoid of 6$\text {\,m}$ internal diameter that can provide a nominal magnetic field of 3.8$\text {\,T}$. Within the solenoid volume in the central region are a silicon pixel and strip tracker, a lead tungstate crystal electromagnetic calorimeter, and a brass and scintillator hadron calorimeter. Muons are measured in gas-ionisation detectors embedded in the steel return yoke. The central detectors of CMS are complemented by calorimeters in the forward direction, which all rely on the detection of Cherenkov photons produced when charged particles pass through their active quartz components. The “hadron forward” (HF) calorimeters cover the pseudorapidity interval $3.0<|\eta |<5.2$ and use quartz fibres embedded in a steel absorber. The CASTOR calorimeter is a sampling calorimeter composed of layers of fused silica quartz plates and tungsten absorbers. It is located on only one side of CMS and covers the region $-6.6<\eta <-5.2$. CASTOR is segmented into 16 azimuthal towers, each with 14 longitudinal channels. The two front channels have a combined depth of 20 radiation lengths and form the electromagnetic section of each tower. The remaining 12 channels constitute the hadronic section. The full depth of a tower amounts to 10 hadronic interaction lengths. A more detailed description of the CMS detector, together with a definition of the coordinate system used and all relevant kinematic variables, can be found in Ref. [17]. A detailed description of the CASTOR calorimeter is given in Refs. [7, 9, 18]. For triggering purposes, the Beam Pickup Timing for the eXperiment (BPTX) devices were used [19].

The data are compared to a broad range of model predictions covering different parameter tunes as well as entirely different physics approaches. The models considered are pythia 8 [20] (version 8.212) with tune CUETP8M1 [21], and tune 4C [3], combined with the MBR [22] model to describe diffractive processes. The data are also compared to the predictions of epos lhc [23] and sibyll 2.1 [24]. For these models, a detailed Monte Carlo simulation of the CMS detector response is performed with the Geant4  [25] toolkit. The simulated events are processed and reconstructed in the same way as the collision data. Furthermore, predictions by QGSJetII.04 [26], sibyll 2.3c [27], pythia 8 tune CP5 [28], and herwig  7.1 [29, 30] with the default tune for soft interactions [31] are also compared to the data. These simulations are produced only at the generator level. A forward folding method is developed to compare generator-level simulations to the data. This technique can be used to compare any model or theoretical prediction to the data and will be described in detail.

## Data analysis and systematic uncertainties

This analysis is based on data recorded during the low-luminosity startup operation of the LHC in June 2015, at a proton-proton centre-of-mass energy of 13$\,\text {TeV}$. In this period the CMS solenoid was turned off. The data correspond to an integrated luminosity of 0.22$\,\text {nb}^{-1}$, with an average proton-proton interaction probability of about 30% per bunch crossing.

The event selection criteria are optimised to select inelastic collision events with minimal bias. The residual contribution of electronic noise and beam background in these events is well below 1%. Events were selected online with an unbiased trigger requiring only the presence of two colliding bunches. The offline event selection requires activity in the HF calorimeters: at least one tower with reconstructed energy larger than 5$\,\text {GeV}$ in either the positive or negative HF calorimeter. In addition, at least one reconstructed track with $|\eta |<2$ is required in the CMS pixel detector. A modified tracking algorithm from Ref. [32] is used in the absence of a magnetic field. Information from the pixel detector is used to reconstruct straight tracks. Signals in all three layers of the pixel detector are required to lie within a cone of radius $R = \sqrt{\smash [b]{(\Delta \phi )^2+(\Delta \eta )^2}} = 0.02$ (where $\phi$ is the azimuthal angle in radians) around the reconstructed track. The efficiency to find more than two hits in the pixel detector drops quickly for $|\eta |>2$; the search for tracks is therefore limited to $|\eta |<2$. Tracks are retained if they originate from the expected interaction region and are linked to at least one interaction vertex. This pixel track reconstruction has an efficiency of about 76% and a probability of $\approx$5% of spurious tracks for charged particles with a transverse momentum $p_{\mathrm {T}}$ larger than 200$\,\text {MeV}$.

To reject events with more than one simultaneous proton-proton interaction (pileup), an additional constraint on the reconstructed interaction vertices is applied. Events with two reconstructed vertices are rejected if the vertices are separated by more than 0.5$\,\text {cm}$ along the *z* axis. This minimises the rejection of events with high particle multiplicity, where the reconstruction may create multiple spurious vertices. The probabilities for events to have additional collisions is evaluated in both data and simulation to be 1.5% (visible vertex) and 2.3% (invisible vertex). The correction of these background events is not straightforward, since the correction depends on the track multiplicity in the central region as well as on the model used in simulation. Therefore, the contribution from pileup events to the forward energy is considered part of the systematic uncertainty of the measurement.

The total energy deposited in CASTOR is obtained by summing the energy measured in each calorimeter tower above the noise threshold, which is determined independently for each tower and varies between 2 and 2.5$\,\text {GeV}$. On average, 76% of the showers due to single electrons or photons are contained within the electromagnetic section of CASTOR, and single hadrons are 71% contained in the hadronic section. Moreover, for a given particle energy, the energies deposited by hadron-induced showers are smaller than electron-induced showers, which is known as noncompensation. These properties were precisely measured with a test beam and are implemented in the detector simulation. It was previously shown that the energy deposited in the corresponding sections of CASTOR can serve as good estimators for the particle-level energy of electrons/photons and hadrons [9]. The electromagnetic and hadronic energies of a given event are defined as the energies deposited in the corresponding detector sections of CASTOR, and the total energy as the sum of both.

The events are classified according to the number of reconstructed charged tracks from the vertex. The average total, electromagnetic, and hadronic energy per event is calculated for each track multiplicity bin. The present data make it possible to study track multiplicities up to 150. The statistical uncertainties of the energy measurement are below 2%, much smaller than the systematic uncertainties. The most important sources of systematic uncertainties are described in the following and are summarised in Table 1:

*CASTOR energy scale* The energy scale uncertainty of CASTOR is 17% [9]. The energy scale is determined using a calibration procedure based on SPS test-beam data, LHC beam halo muon events, a cross-calibration to the HF calorimeters, and LED test pulses, in combination with a precise detector alignment. The precision is currently limited by systematic effects related to the modelling and understanding of particle shower cascades in the calorimeter ranging from GeV to TeV energies.

*CASTOR intercalibration* The relative intercalibration is performed using the measured response of each channel to single LHC beam halo muon events, which were recorded with a dedicated trigger during LHC interfill periods. This procedure is limited by the available muon statistics. For a measurement of the total energy, the uncertainty caused by intercalibration is averaged over the whole calorimeter and is 2–3%. For the determination of the electromagnetic and hadronic energy fractions, on the other hand, the effect of relative calibration becomes more significant. Dedicated studies based on full detector simulations of collision events demonstrate that the observed average shape of the longitudinal shower absorption in the calorimeter is consistent with only a slight overestimation of electromagnetic energies, and a corresponding underestimation of hadronic energies. We determine a maximum decrease of the electromagnetic energy by 8% and a corresponding increase of the hadronic energy by 15%, which are included as systematic uncertainties.

*Pileup rejection* The uncertainty arising from the pileup contribution is estimated by considering alternative vertex multiplicity selections; events with exactly one reconstructed vertex, as well as events with two vertices separated by less than 0.7$\,\text {cm}$, are selected. These changes mainly affect the high-multiplicity region and lead to a systematic energy uncertainty of up to 10% for multiplicity >140. Collisions that do not create visible vertices in the detector introduce an additional uncertainty that is below 0.8%.

*HF energy scale* The uncertainty in the reconstructed HF energies is 10% [33]. Varying the threshold for the event selection from 5.0$\,\text {GeV}$ per HF calorimeter tower to 4.5 and 5.5$\,\text {GeV}$ changes the average energy observed in CASTOR by less than 0.5%.

*Tracking* The track reconstruction uncertainty has been previously determined from studies comparing data and simulation [32]. The uncertainties in the tracking and vertexing efficiencies affect the number of reconstructed tracks by 1.8 and 2–3%, respectively. These are combined linearly, yielding a 5% systematic uncertainty in the number of reconstructed tracks. The effect in the average energy is below 5%.

Most of the uncertainties described here are uncorrelated and are therefore added in quadrature. Moreover, in the measured ratios between electromagnetic and hadronic energies the absolute energy scale uncertainty cancels, while the intercalibration uncertainty introduces a particular anticorrelated effect since a systematic decrease of the electromagnetic energy causes an increase of the hadronic energy and vice versa.

**Table 1:** Uncertainties in the average energies measured with the CASTOR calorimeter at the detector level. Ranges indicate the variation as a function of the track multiplicity

Source	Total energy (%)	Electromagnetic energy (%)	Hadronic energy (%)
CASTOR energy scale	17	17	17
CASTOR intercalibration	2–3	−8	$+$15
HF energy scale	<0.5	<0.5	<0.5
Track reconstruction	1–5	1–5	1–5
Pileup rejection	1–8	1–8	1–10
Statistical uncertainty	0.05–1.6	0.06–1.9	0.06–1.8
Total	18–19	18–20	20–26

## Forward folding of model predictions

The measured track multiplicity is distorted with respect to the true charged particle multiplicity by the effects of acceptance and efficiency of the CMS pixel tracker. Likewise, the energies observed in CASTOR are affected by the energy resolution and the response of the calorimeter. In the present paper, the data are not corrected for these effects, and should thus be compared to the results of a full Monte Carlo detector simulation to compare with other experimental data and to future model predictions. For this purpose, a “forward folding” approach is used here, in which all known detector effects are applied to a given model prediction or theoretical calculation. The forward folding approach is chosen since it yields better systematic uncertainties compared to an unfolding of these data.

At the generator level, events are selected that match the detector-level event selection. At least one charged particle with $p_{\mathrm {T}} >200\,\text {MeV}$ is required within $|\eta |< 2$. Furthermore, a fractional momentum loss of the scattered proton of $\xi > 10^{-6}$ is required. To determine $\xi$ all stable ($c\tau >1\,\text {cm}$) final-state particles are divided into two systems, X and Y, based on their position with respect to the largest rapidity gap in the event. All particles on the negative side of the largest gap are assigned to system X, while the particles on the positive side are assigned to system Y. Based on this, we determine $\xi =\max \left( M_{\mathrm {X}}^2/s,\,M_{\mathrm {Y}}^2/s\right)$, where $M_{\mathrm {X}}$ and $M_{\mathrm {Y}}$ are the invariant masses of the two systems. The selection based on $\xi$ is relevant at very low particle multiplicities, and leads to an optimal agreement with the event selection as implemented at the detector level. It is also consistent with previous CMS publications, e.g. Ref. [9, 34].

Four-dimensional migration matrices *k* describing the probability to reconstruct an event with central multiplicity $N_{\mathrm {tracks}}$ and forward energy $E_{\mathrm {reco}}$ for given values $N_{\mathrm {ch}}$ and $E_{\mathrm {true}}$ are calculated based on all available Monte Carlo samples with full detector simulation. At the generator level, the central multiplicity $N_{\mathrm {ch}}$ is defined as the number of stable charged final-state particles with $p_{\mathrm {T}} >200$ $\,\text {MeV}$ and $|\eta |< 2$, and the forward energy $E_{\mathrm {true}}$ is defined as the sum of the energies of all particles within $-6.6<\eta <-5.2$ except for muons and neutrinos. At the detector level, the number of reconstructed tracks with $|\eta |<2$ is $N_{\mathrm {tracks}}$ and the reconstructed energy in CASTOR is $E_{\mathrm {reco}}$. The four-dimensional matrices $k_{ij}^{lm}$ are constructed with 20 bins in $N_{\mathrm {ch}}$ and $N_{\mathrm {tracks}}$ ranging from 1 to 200 (dimensions *i* and *l*) , as well as 46 bins in $E_{\mathrm {true}}$ and $E_{\mathrm {reco}}$ ranging from 0 to 10$\,\text {TeV}$ (dimensions *j* and *m*). The bin intervals used at detector and generator level are identical. The range of *k* is larger than that used for the final results in order to allow for the effects of bin migration. Final results are presented for $N_{\mathrm {tracks}}$ between 1 and 150.

All four components of *k* have one extra underflow bin to handle the event selection efficiency. If an event does not pass the event selection criteria at the generator level ($N_{\mathrm {ch}}\ge 1$ and $\xi >10^{-6}$), it is recorded in the underflow region with $N_{\mathrm {ch}}=0$ and $E_{\mathrm {true}}=-1\,\text {GeV}$. If an event is not selected at the detector level (one HF tower above 5$\,\text {GeV}$ and $N_{\mathrm {tracks}}\ge 1$), it is recorded in the underflow region with $N_{\mathrm {tracks}}=0$ and $E_{\mathrm {reco}}=-1\,\text {GeV}$. In this way, the effects of inefficiencies and migrations from outside the visible phase space are included in *k*. For example, the selection efficiency for events having a specific $N_{\text {ch}}$ and $E_{\text {true}}$ is the ratio of the number of events without the underflow bin to the number of events with the underflow bin.

Two-dimensional distributions, $N_{\text {reco}}^{ij}$, describing the event yields in bins (*i*, *j*) of $N_{\mathrm {tracks}}$ and $E_{\mathrm {reco}}$ can then be obtained for any given event generator or theoretical prediction by means of the following matrix multiplication:

1 $$\begin{aligned} N_{\text {reco}}^{ij} = \sum _{l,m} k_{ij}^{lm} N_{\text {true}}^{lm}, \end{aligned}$$

where $N_{\text {true}}^{lm}$ is the distribution of generator-level events in bins (*l*, *m*) of $N_{\text {ch}}$ and $E_{\text {true}}$. The average energy in each track multiplicity bin is calculated from $N_{\text {reco}}^{ij}$ excluding the underflow bins, and is compared to the data directly at the detector level. The results obtained by using the forward folding method coincide with those obtained with the full detector simulation to better than 1%.

The matrix *k* has a slight dependence on the $\eta$, $p_{\mathrm {T}}$ and multiplicity distributions of the final-state particles in the event generator used in the full detector simulation. To quantify this dependence, four matrices are provided based on pythia 8 tune CUETP8M1, pythia 8 tune 4C+MBR, epos lhc, and sibyll 2.1. A fifth matrix is obtained by averaging the matrices of these models and serves as the central value for all forward-folded results. The spread of the results obtained with the individual matrices is an estimate of the systematic uncertainty related to the model dependence; it is mostly well below 5%, but reaches 15% in a few bins. All five variations of *k* are available in a rivet [35] plugin. This way, the forward folding can be applied to any other model prediction. Moreover, the full point-to-point correlation of the model-related uncertainty can be studied.

## Results

**Fig. 1:**
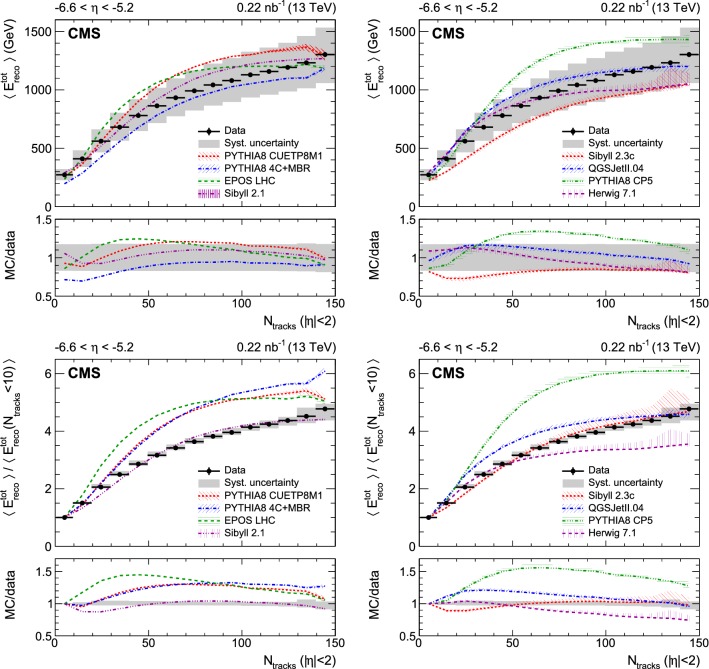
Top panel: Average total energy reconstructed in the CASTOR calorimeter as a function of the number of reconstructed tracks for $|\eta |<2$. Bottom panel: Average total energy reconstructed in the CASTOR calorimeter normalised to that in the first bin ($N_{\mathrm {ch}}<10$) as a function of the number of reconstructed tracks for $|\eta |<2$. In all figures, the data are shown as black circles and the corresponding systematic uncertainties with a gray band; horizontal bars are used to indicate the bin width. The predictions of various event generators are compared to the data, which are the same in both panels. The bands associated with the model predictions illustrate the model uncertainty

**Fig. 2:**
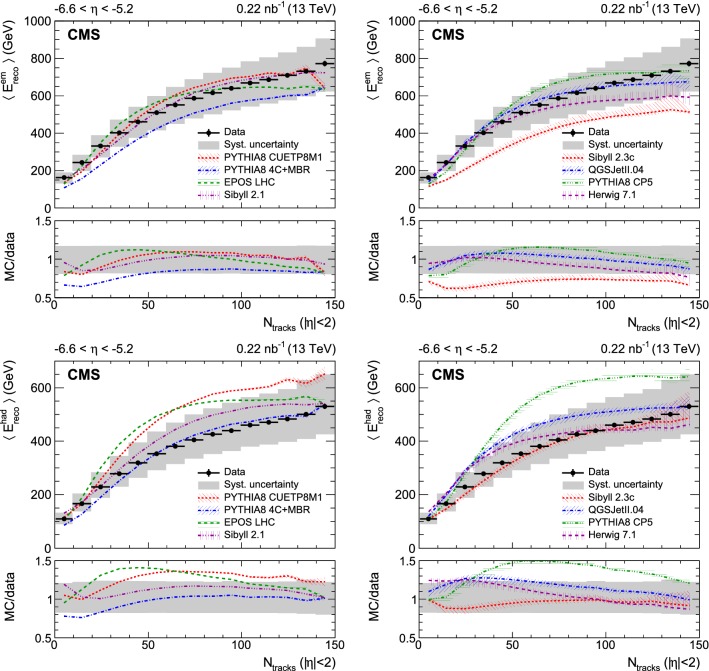
Top panel: Average electromagnetic energy reconstructed in the CASTOR calorimeter as a function of the number of reconstructed tracks for $|\eta |<2$. Bottom panel: Average hadronic energy reconstructed in the CASTOR calorimeter as a function of the number of reconstructed tracks for $|\eta |<2$. In all figures, the data are shown with black circles and the corresponding systematic uncertainties with a gray band; horizontal bars are used to indicate the bin width. The predictions of various event generators are compared to the data, which are the same in both panels. The bands associated with the model predictions illustrate the model uncertainty

**Fig. 3:**
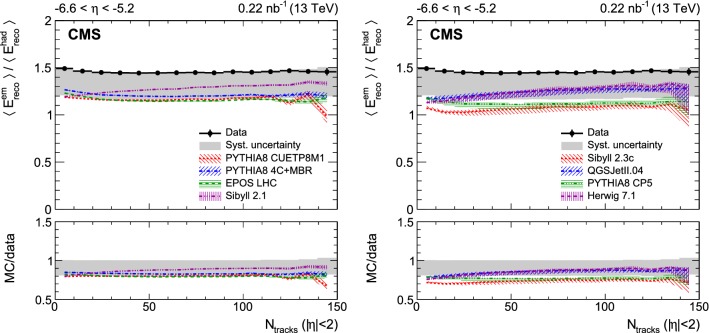
Ratio of average electromagnetic and hadronic energies reconstructed in the CASTOR calorimeter as a function of the number of reconstructed tracks for $|\eta |<2$. The data are shown with black circles and the corresponding systematic uncertainties with a gray band; horizontal bars are used to indicate the bin width. Predictions of various event generators are compared to the data, which are the same in both panels. The bands associated with the model predictions illustrate the model uncertainty

Various measurements of the average energy reconstructed in the region $-6.6<\eta <-5.2$ are presented as a function of the track multiplicity for $|\eta |<2$ in Figs. 1, 2, and 3. The statistical uncertainties of the data are small and therefore not visible. The systematic uncertainties are shown with a gray band. The data are not corrected for detector effects and are compared to the predictions of models commonly used to describe hadron interactions at the LHC and in high energy cosmic ray air showers. These models are grouped into two sets:

The first contains pythia 8 tune CUETP8M1 and tune 4C+MBR, epos lhc and sibyll 2.1. All these have a full detector simulation. The error bands shown for these models reflect only the Monte Carlo statistical uncertainties. These become visible especially in the last bin.

The second set of models consists of sibyll 2.3c, QGSJetII.04, pythia 8 tune CP5, and herwig  7.1. Predictions from these models are obtained using the forward-folding method. The uncertainty bands shown for these models also include the systematic uncertainties from the forward-folding procedure discussed in the previous section.

The average total energy in CASTOR, shown in Fig. 1 (upper), increases with the track multiplicity. This feature is consistent with the general behaviour of the underlying event measured at central rapidities (see for example Refs. [10, 11]) and is reproduced by all models. The rise can be associated to an initial correlation of central and forward event activity, which is damped by energy conservation in the most violent collisions. All models describe these data with at most minor discrepancies. This implies that the model parameters for the underlying event determined at central rapidities are valid also for the very forward data. In detail, the energies predicted by pythia 8 4C+MBR and sibyll 2.3c are slightly too low at small multiplicity. Conversely, at intermediate multiplicities, pythia 8 CP5 predicts average energies larger than those observed.

The systematic uncertainty in the data is dominated by the energy scale uncertainty contribution, which is fully correlated between the multiplicity bins. Therefore, the distributions can be normalised to the first bin, so that, when comparing their shapes, the systematic uncertainty is significantly smaller (cf. Fig. 1, lower). The rise is steep at low multiplicities and becomes more gradual at higher multiplicities. All pythia 8 tunes have very similar shapes, inconsistent with that observed in the data. The disagreement is strongest for pythia 8 CP5, a tune optimised on underlying event data at central rapidity. This tune uses parton distribution functions at next-to-next-to-leading order and features a softer MPI cutoff compared to pythia 8 CUETP8M1 (see Ref. [28] for details). The data therefore provide relevant information for future generator improvements and tunes. The epos lhc, QGSJetII.04, and herwig 7.1 models predict saturation at multiplicities above 80, which is not seen in the data. Both versions of sibyll provide predictions in agreement with the data.

The individual electromagnetic and hadronic energy distributions are shown in Figs. 2 (upper) and 2 (lower). All models, with the exception of sibyll 2.3c, describe the electromagnetic component well. pythia 8 4C+MBR slightly underestimates the electromagnetic energy at low multiplicities. Conversely, the other models tend to overestimate the hadronic component. Specifically these data can be very relevant for improving the simulation of cosmic ray induced extensive air showers, and specifically the modelling of the production of neutral versus charged pions or other hadrons with longer lifetimes, since the energies in the region $-6.6<\eta <-5.2$ are close to those in the peak of the forward energy flow.

The data are also used to determine the ratio of the average electromagnetic and hadronic energies (Fig. 3). Here, the relative calibration of the electromagnetic and hadronic sections is the main source of uncertainty and results in a very asymmetric uncertainty band. The measured ratio is approximately constant over the whole multiplicity range. The ratio is sensitive to the details of hadronisation, and discrepancies between models and data may reflect an inadequate description of the hadron production mechanisms. String fragmentation, remnant fragmentation, initial- or final-state radiation, the effects of a possible very dense hydrodynamical phase, or the decay of short-lived resonances may be relevant to the understanding of the data. The observed independence of the measured ratio of track multiplicity indicates that no dramatic change of the particle production mechanism is observed at this very forward pseudorapidity. All model predictions are lower than the data, specifically those of the modern tunes pythia 8 CP5 and sibyll 2.3c, whereas QGSJetII.04, sibyll 2.1, and herwig 7.1 provide the best description of the ratio.

## Summary and discussion

The average energy per event in the pseudorapidity region $-6.6<\eta <-5.2$ was measured as a function of the observed central track multiplicity ($|\eta |<2$) in proton-proton collisions at a centre-of-mass energy of 13$\,\text {TeV}$. The data are recorded during the first days of 13$\,\text {TeV}$ running with low beam intensities. The measurement is presented in terms of the total energy as well as its electromagnetic and hadronic components. The very forward region covered by the data contains the highest energy densities studied in proton-proton collisions at the LHC so far. This makes the present data relevant for improving the modelling of multiparticle production in event generators of ultra-high energy cosmic ray air showers.

The measured average total energy as a function of the track multiplicity is described by all models reasonably well. This demonstrates that the underlying event parameter tunes determined at central rapidity can be safely extrapolated to the very forward region within experimental uncertainties. A shape analysis indicates, however, that there are significant differences among the models and large deviations from the data. The generator sibyll 2.1 gives the best description of the measured multiplicity dependence of the average total energy.

The data are also presented in terms of the average electromagnetic and hadronic energies per event as a function of the central track multiplicity. This is useful in the study of different particle production mechanisms, since the former is primarily due to the decay of neutral pions and the latter to the production of hadrons with longer lifetimes, mostly charged pions. All models give a good description of the electromagnetic energy dependence on the multiplicity, with the exception of sibyll 2.3c. Conversely, the predictions for the hadronic energy have a significantly larger spread compared to the electromagnetic case.

The ratio between the electromagnetic and hadronic energies is also presented. The data exhibit a larger fraction of electromagnetic energy than the models, and disagree with the two most recent model tunes, i.e. sibyll 2.3c and pythia 8 CP5. Therefore, these models cannot explain the muon deficit in ultra-high energy air shower simulations since the data indicate that even more energy must be channelled into the electromagnetic part of the cascade and is thus lost for the generation of further hadrons [16].

## Data Availability

This manuscript has no associated data or the data will not be deposited. [Authors’ comment: Release and preservation of data used by the CMS Collaboration as the basis for publications is guided by the CMS policy as written in its document “CMS data preservation, re-use and open access policy” (https://cms-docdb.cern.ch/cgi-bin/PublicDocDB/RetrieveFile?docid=6032&filename=CMSDataPolicyV1.2.pdf&version=2 ).]
